# Management of chronic rhinosinusitis with nasal polyps in the Asia-Pacific region and Russia: Recommendations from an expert working group

**DOI:** 10.5415/apallergy.0000000000000139

**Published:** 2024-04-04

**Authors:** Sergey Karpischenko, Yong Gi Jung, Dae-Woo Kim, Kymble Spriggs, Raymond King-Yin Tsang, Te-Huei Yeh

**Affiliations:** 1 ENT Department, Pavlov First Saint Petersburg State Medical University, Saint Petersburg, Russia; 2 ENT Department, K.A. Rauhfus Children’s City Multidisciplinary Clinical Center for High Medical Technologies, Saint Petersburg, Russia; 3 Department of Otorhinolaryngology-Head and Neck Surgery, Samsung Medical Center, Sungkyunkwan University School of Medicine, Seoul, Republic of Korea; 4 Department of Otorhinolaryngology-Head and Neck Surgery, Boramae Medical Center, Seoul National University College of Medicine, Seoul, Republic of Korea; 5 Department of Medicine, The University of Melbourne, Melbourne, Australia; 6 Division of Otolaryngology-Head and Neck Surgery, Department of Surgery, School of Clinical Medicine, The University of Hong Kong, Hong Kong SAR, China; 7 Department of Otolaryngology-Head and Neck Surgery, Yong Loo Lin School of Medicine, National University of Singapore, Singapore; 8 Department of Otolaryngology, National Taiwan University Hospital, Taipei, Taiwan; 9 Department of Otolaryngology, College of Medicine, National Taiwan University, Taipei, Taiwan

**Keywords:** Biologics, chronic rhinosinusitis with nasal polyposis, type 2 inflammation

## Abstract

Chronic rhinosinusitis with nasal polyps (CRSwNP) is a chronic inflammatory condition of the nasal and paranasal tissues, characterized by the presence of bilateral nasal polyps. An expert panel of specialists from the Asian-Pacific region and Russia was convened to develop regional guidance on the management of CRSwNP through a consensus approach. The present article presents the chief observations and recommendations from this panel to provide guidance for clinicians in these areas. Etiology and pathogenetic mechanisms in CRSwNP are heterogeneous and complex. In many patients, CRSwNP is primarily driven by type 2 inflammation, although this may be less important in Asian populations. Frequent comorbidities include asthma and other inflammatory diseases such as non-steroidal anti-inflammatory drug (NSAID)/aspirin-exacerbated respiratory disease or atopic dermatitis. Clinical management of CRSwNP is challenging, and a multidisciplinary approach to evaluation and treatment is recommended. While many patients respond to medical treatment (topical irrigation and intranasal corticosteroids, and adjunctive short-term use of systemic corticosteroids), those with more severe/uncontrolled disease usually require endoscopic sinus surgery (ESS), although outcomes can be unsatisfactory, requiring revision surgery. Biological therapies targeting underlying type 2 inflammation offer additional, effective treatment options in uncontrolled disease, either as an alternative to ESS or for those patients with persistent symptoms despite ESS.

## 1. Introduction

Chronic rhinosinusitis (CRS) is a common often debilitating condition, with a global prevalence of symptomatic disease ranging from 5.5% to 28% [1, 2]. Two broad CRS phenotypes exist, characterized on the basis of the presence or absence of nasal polyps [1, 2]. Patients with chronic rhinosinusitis with nasal polyps (CRSwNP) account for up to 20% of all CRS [3].

The clinical management of CRSwNP can be complex. Conventional treatment approaches include nasal irrigation, topical intranasal corticosteroids (INS) or oral corticosteroids (OCS) [1, 2], and endoscopic sinus surgery (ESS) in patients with persistent or uncontrolled disease [4, 5]. The development and introduction of biologics targeting the underlying inflammatory processes involved in CRSwNP provides additional treatment options for those patients not responding to conventional therapy. Clinical decision-making on the management and treatment of CRSwNP is helped by comprehensive evidence-based specialist guidelines, which continue to evolve and update in response to the latest evidence. Such guidance includes the European Position Paper on Rhinosinusitis and Nasal Polyps (EPOS 2020) [1, 2], consensus-based recommendations from the European Forum for Research and Education in Allergy and Airway Diseases (EUFOREA) [6, 7], and the International Consensus Statement on Allergy and Rhinology: Rhinosinusitis (ICAR-RS-2021) [8].

While useful from an international or global perspective, such guidance may not fully accommodate differences in clinical disease patterns and clinical resources and therapeutic options at a regional level. Development of guidance from a more regional perspective, which may be more aligned with physician experience (and patient needs) in such settings, can provide additional value. At present, such guidance is relatively limited for the Asian-Pacific (APAC) region, although some recent guidelines or position papers have now been published. These include the position paper on CRSwNP management jointly developed by the Australasian Society of Clinical Immunology and Allergy and the Australian Society of Otolaryngology Head and Neck Surgery [9], and the recent guideline on use of nasal irrigation in adults with CRS from the Korean Society of Otorhinolaryngology-Head and Neck Surgery and the Korean Rhinologic Society [10]. At present, such guidance is lacking for Russia. Another consideration is that many patients with CRSwNP are managed chiefly within primary care, and guidance tailored toward a broader physician audience may assist in decision-making and appropriate referral to specialist care.

To develop such guidance across the broader APAC region and for Russia, an expert panel was convened to evaluate the utility of existing guidelines such as EPOS 2020 to clinical practice and to develop guidance for physicians in APAC countries and Russia on the management of CRSwNP. The present article presents the chief observations and recommendations from this panel.

## 2. Methodology

A multidisciplinary expert panel was formed comprising specialists in respiratory allergy and otorhinolaryngology (ORL) from Australia (KS), Hong Kong (RK-YT), the Republic of Korea (YGJ, D-W K), Russia (SK), and Taiwan (T-HY). Panelists were selected based upon experience and expertise in treating CRSwNP, publication record, and involvement in similar activities.

Two online meetings were held, in October and December 2021. The first comprised a general discussion on CRSwNP disease and management within the APAC region and Russia and on the utility of available global or regional guidance. The second meeting focused specifically on the recommendations and underlying supportive rationale for adult CRSwNP reported within the EPOS 2020 guidelines [1, 2], with discussion on existing gaps or possible modifications to address the loco-regional context and needs. A series of discussion points and recommendation proposals for across a range of topics were then developed and reviewed using an interactive online platform (Within3; https://www.within3.com), which provides the opportunity for communication within the panel to explain their decision-making. In this step (conducted between January 31 and February 14, 2022), each panel member indicated their agreement or disagreement to each discussion point or proposed recommended statement in turn (indicating any necessary changes and provided their reasoning where necessary). Note that the aim was not to generate specific formal recommendation statements (or to report on any consensus agreement values). The discussion points, specific feedback, and any dialog between panelists were collated, and then presented and structured as a series of explanatory text, recommendations (and supportive rationale). This formed the basis of an early draft of the present manuscript. All panelists then reviewed and commented on manuscript drafts in successive rounds, with approval of the final version considered as final agreement for the present guidance on the management of CRSwNP. This consensus exercise was conducted through review of publicly available literature and did not involve specific human participants or any identifiable data; institutional review board approval was not required.

## 3. Disease definitions and diagnostic approach

There are clear diagnostic criteria for rhinosinusitis, CRS, and CRSwNP based upon clinical symptoms and evidence of sinonasal mucosal disease. Diagnosis of acute rhinosinusitis requires the presence of ≥2 symptoms, one of which should be either nasal blockage, obstruction, congestion, or discharge, and either facial pain/pressure, headache and/or reduction, or loss of smell [1, 2]. Confirmation of mucosal disease (presence of polyps, mucopurulent discharge and/or edema, or mucosal obstruction within the ostiomeatal complex or sinuses) via endoscopy or computed tomography (CT) is also required [1, 2]. Although in routine clinical practice, acute rhinosinusitis may be diagnosed on clinical grounds alone, the importance of endoscopic/CT confirmation of CRS (and for CRSwNP) must be emphasized, as endoscopic/CT findings dictate disease classification and subsequent treatment planning. As discussed below, early referral for specialist evaluation and management of CRS (and CRSwNP) is essential.

The cardinal symptoms of CRS are similar to acute disease, that is, nasal obstruction, congestion or discharge, facial pain/pressure, and olfactory disturbance/loss of smell [1, 2, 11, 12]. Overt facial pain with CRSwNP is uncommon in the Asian patients, where headache (or complaints of pressure or fullness) or nasal obstruction may be more common presenting symptoms. While allergic symptoms (eg, sneezing or itching) are not part of existing diagnostic criteria, their presence may indicate an additional diagnosis of allergic rhinitis. In EPOS 2020, the diagnosis of CRS requires rhinosinusitis lasting for ≥12 weeks (without interruption, as patients may experience recurrent episodes of acute rhinosinusitis) [1, 2]. This assumes that any acute rhinosinusitis has been managed appropriately (ie, with nasal irrigation and INS). Diagnosis of CRSwNP requires the presence of bilateral, endoscopically visualized polyps in the middle meatus [1, 2]. While a minimum duration of 12 weeks reflects a useful policy for chronic disease diagnosis from a symptomatic perspective, this need not be considered absolute. For example, if evidence of more chronic and significant disease already exists (eg, objective findings of nasal polyps on endoscopic examination), then an earlier presumptive diagnosis of CRSwNP would seem appropriate. As discussed later, what is perhaps most important is the need for timely referral for specialist evaluation at the earliest opportunity. Where polyps are absent, patients are considered to have chronic rhinosinusitis without nasal polyps (CRSsNP). When endoscopy or CT shows only unilateral disease, then alternative diagnoses (especially neoplasia) must be considered [1, 2].

## 4. Etiology and pathogenesis

Pathophysiologic mechanisms in CRSwNP involve a complex interaction of host and environmental factors, where specific individual patient profiles are a product of both endotype and phenotype. CRS and specific types such as CRSwNP are now characterized on the basis of the predominant underlying inflammatory pattern (or endotype) and predominant clinical phenotype in current classifications such as EPOS 2020 [1, 2]. Endotypes broadly reflect the underlying biology and pathogenetic mechanisms such as the inflammatory profile (eg, a type 2 dominant inflammatory signature), now especially relevant following the development of novel biologic therapies targeting underlying type 2 inflammatory processes in CRSwNP [13, 14]. Phenotypes reflect the patient’s clinical profile (including comorbid disease) [15, 16]. From a phenotypic perspective, the broad global pattern is that while asthma is more prevalent in patients with CRS compared with the general population, coexisting asthma is even more strongly associated with CRSwNP (30%–70% of patients) and the severity of asthma is also greater in CRSwNP [3, 17-19]. Comorbidities such as allergic rhinitis, NSAID/aspirin-exacerbated respiratory disease, and atopic dermatitis are also more prevalent in CRSwNP than in CRSsNP [20]. This association of CRSwNP and other inflammatory disease often reflects the common underlying type 2 inflammatory processes in these conditions, with eosinophilic tissue inflammation mediated by type 2 cytokines, for example, interleukin (IL)-4, IL-5, and IL-13 and local/circulating IgE, observed in the majority of patients with CRSwNP [15]. However, these patterns show substantial heterogeneity in different geographical populations (including within the APAC region). For example, in Hong Kong, only approximately one-third of CRSwNP patients have comorbid asthma. In Australia, while the majority of CRSwNP patients show a type 2 inflammatory endotype, in countries such as South Korea and Taiwan, a nontype 2 endotype is more prevalent [21], and where mucosal barrier dysfunction may play a more important role in CRSwNP development.

## 5. CRS classification

In EPOS 2020, CRS is classified firstly on the basis of disease distribution, that is, localized (unilateral) and diffuse (bilateral), and then on the basis of the dominant endotype (eg, the presence/absence of type 2 inflammation) and the clinical phenotype [1, 2]. Diffuse CRS disease with predominantly type 2 inflammatory endotype includes CRSwNP (although other conditions with predominantly type 2 inflammatory pathways are also included within this category). These include allergic fungal rhinosinusitis (AFRS) and central compartment atopic disease (CCAD), a relatively recently described variant of CRS associated with inhalant allergen exposure [1]. While both AFRS and CCAD are characterized by the presence of polyps, these conditions are classified separately from CRSwNP [1, 2]. AFRS can be distinguished from CRSwNP by the presence of eosinophilic mucin with noninvasive fungal hyphae within the sinonasal mucosa, and often (but not always) a type I hypersensitivity reaction to fungi [2, 22, 23]. In CCAD, polypoid mucosal changes are observed, and while this may seem similar to CRSwNP on CT, these can be differentiated on CT and nasal endoscopy [24].

To some extent, the focus on CRSwNP in EPOS 2020 is principally in the context of disease driven by type 2 inflammation (reflecting the greater importance in western patient populations). As indicated earlier, while the majority of CRSwNP patients in Australia present with a type 2 inflammatory profile, this is less commonly seen in in South Korea and Taiwan, where a type 2 inflammatory signature may be absent (with the inflammatory profile principally being either type 1 or type 3). There remains a need to develop guidelines that focus on CRSwNP with a nontype 2 inflammatory endotype (and the role of biologics in these patients).

## 6. Care pathways and diagnostic and clinical assessment

Multidisciplinary specialist management involving both ORLs and allergists/immunologists/pulmonologists provides an optimal approach to ensure comprehensive evaluation and optimal use of the most appropriate surgical and medical therapeutic strategies [25, 26]. For CRS, immediate referral is essential in the presence of any red-flag alarm signs or symptoms (eg, visual upset, severe headache, and neurological signs) [1, 2]. Although EPOS 2022 describes a self-care element (in which patients may receive advice and over the counter symptomatic relief medication), the panel recommends early referral from primary care for all patients with CRS or suspected CRSwNP (ideally within 6–12 weeks) [1, 2]. This facilitates earlier endoscopic and/or CT assessments necessary to confirm CRSwNP and exclude other diagnoses, and also the more complete clinical and laboratory evaluation to help define the associated inflammatory endotype and phenotype (including relevant comorbidities).

Clinical and laboratory evaluation of CRSwNP follows well-established guidance [1, 2, 6-8, 27]. Both endoscopy and CT have high diagnostic accuracy [2, 28], although choice may differ depending on country and physician specialty. Endoscopy is often preferred by ORL specialists. CT scans may be initially used by physicians in other specialties (and is useful in excluding alternative diagnoses) and in the ORL setting often in later stages of clinical management (eg, in surgical evaluations). For both endoscopy and CT, the extent of sinus disease can be readily assessed using simple commonly used measures [1, 6, 7]. The endoscopic nasal polyp score (NPS) assesses polyp size, number, and extent of sinus involvement to generate a total NPS ranging from 0 to 8, with higher scores indicating more extensive disease [29]. CT evaluation via the Lund–Mackay scoring system (LMS) is long-established (and widely used as an outcome in clinical studies) [30]. This grades each of the paranasal sinuses the basis of no, partial, or complete opacification (scored as 0, 1, or 2, respectively) to generate a total LMS score ranging from 0 to 24 [30].

Laboratory investigations include assessment of total serum IgE and serum eosinophils, each of which are important biomarkers to assess endotype and align with the presence of type 2 inflammation. Although tissue eosinophilia is also useful, this is often performed on surgical specimens and may not necessarily be part of the routine initial workup. While specific criteria for biomarkers supportive of a type 2 inflammatory endotype in the context of APAC countries are discussed later, when possible, evaluation of these markers at the earliest opportunity is preferred (and ideally before any significant systemic corticosteroid use). This can inform the broader treatment strategy (eg, suitability for biologics) at an early stage of patient management. A complete assessment of comorbidities is also essential. Consultation with pulmonologists is recommended for CRSwNP patients with comorbid asthma, as this may influence treatment choices and outcomes [7, 26].

In EPOS 2020, CRSwNP disease severity is based upon assessment of disease impact on general quality of life (QoL) [2]. The Sino-Nasal Outcome Test-22 (SNOT-22) is the most widely used tool [31, 32]. SNOT-22 is a 22-item patient questionnaire that asks the patient to rate severity/impact of physical symptoms and impact on health-related QoL to generate a total SNOT-22 score (range, 0–110). Scores >50 reflect severe disease impact [31, 32]. When used as measure to assess treatment effects, a reduction of 8 to 9 points is considered as the minimum clinically important difference in the SNOT-22 score [33, 34].

Physician assessment of control is chiefly based upon improvement or stabilization of clinical findings and endoscopic examination, while patient-reported symptom severity, disturbance in smell, and impact on QoL and sleep or fatigue can be measured via a visual analog scale or the SNOT-22 questionnaire. Assessment of olfactory impairment is important, although patient self-evaluation of is highly subjective. Objective assessment can be made using a variety of tests such as the University of Pennsylvania Smell Identification Test, although this has well-recognized cultural biases [2], and may not necessarily be practical for routine olfactory testing in the APAC region.

## 7. Treatment approach

Goals of CRSwNP treatment are to achieve and maintain clinical control (whereby following treatment the patient is symptom-free or where symptoms are not impacting QoL). The EPOS 2020 guidelines consider CRSwNP disease control based on the presence/absence of clinical signs and symptoms; nasal blockage, mucopurulent rhinorrhea/postnasal drip, facial pain/pressure, smell impairment, sleep disturbance or fatigue, the presence/absence of mucosal disease on endoscopy, and use or need for rescue medication, that is, short-term OCS or antibiotics [1, 2]. In this approach, when none of these are present, CRSwNP can be considered controlled. Where 1 of these findings is present, CRSwNP is considered as partly controlled, while if 3 or more of these findings exist, the disease is considered to be uncontrolled [1, 2]. While this standardized approach has some value, especially in the research setting, the panel’s view is that in routine clinical care, the persistence of symptoms despite appropriate treatment (or of continued mucosal disease findings on endoscopy even in the absence of symptoms) is perhaps a more useful guide to considering disease control. In this approach, CRSwNP may be considered as uncontrolled if one or more of the above signs or symptoms persist despite appropriate treatment, with the need for continued or alternative treatment. This approach aligns with the most recent EUFOREA guidance [7].

## 8. Principles of treatment

**Figure 1.**
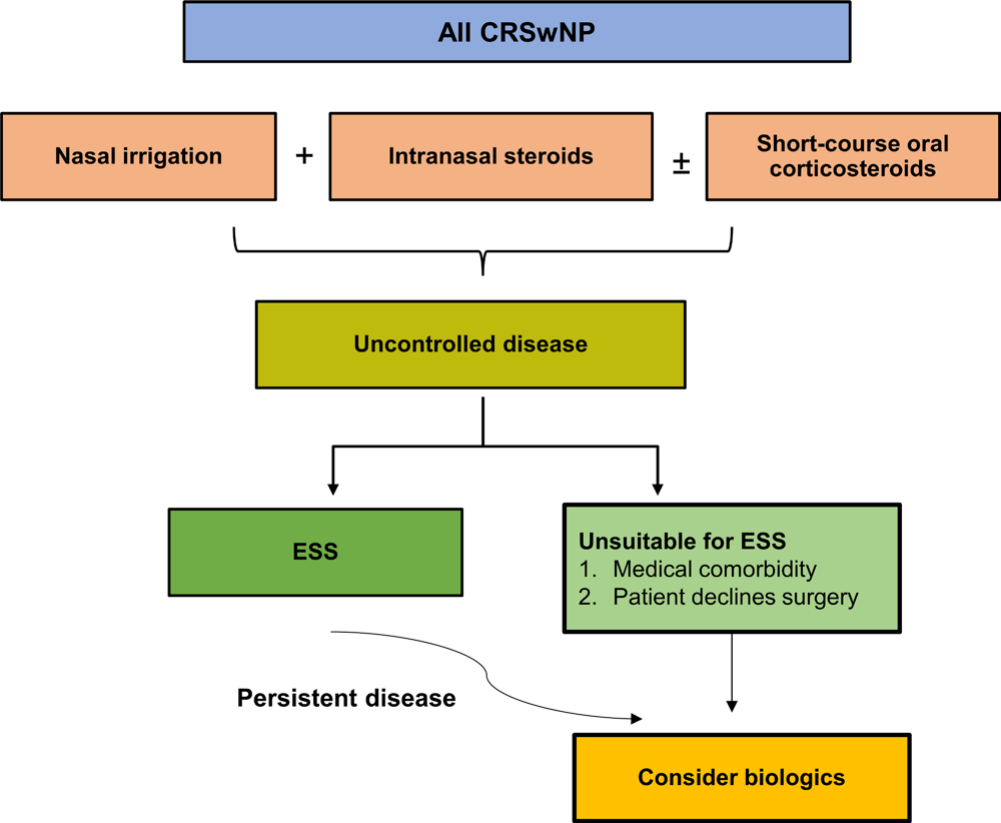
Step-wise approach to treatment in CRSwNP. Initial first-line medical therapies include nasal irrigation, intranasal steroids, and short-term oral corticosteroids. Patients with uncontrolled disease require complete ESS (unless considered unsuitable). Biologics can be considered in those patients with persistent disease following ESS. Biologics may also be considered as an alternative to ESS in individuals who are considered unsuitable or who decline surgery. First-line therapies should be continued following surgery and in conjunction with biologics. CRSwNP, chronic rhinosinusitis with nasal polyps; ESS, endoscopic sinus surgery.

From a treatment perspective, principal first-line options are nasal saline irrigation and use of topical INS, either as sprays or drops, and intermittent use of OCS [1, 2, 9] (Fig. 1). Topical nasal irrigation is a key element of CRSwNP treatment, and practical advice on self-care (ie, correct nasal irrigation technique) is fundamental. While different solutions may be used, isotonic saline is a safe and effective and convenient low-cost option [10]. A wide range of INS preparations are available [20, 35-37], and INS are well tolerated with no significant adverse effects [1, 2, 9]. During EPOS 2020 guideline development, the steering group appraised those studies evaluating INS and concluded that there was evidence for some improvement in CRSwNP symptoms and in QoL (with reductions in SNOT-22 scores) [1, 2]. Although antibiotics are often used, there are limited data for benefit in the treatment of CRSwNP [1, 2]. If response is poor, OCS can be used, although only as short-term use (eg, for up to 2 weeks) due to recognized risks with prolonged treatment [1, 2, 38]. There is some evidence for short-term improvement in symptoms (eg, smell and nasal blockage) with OCS [1, 2, 39], and short-term intermittent use (eg, 1–2 courses per year) can be beneficial in patients with poor response or uncontrolled disease [1, 2]. Some patients may be reluctant to use OCS, an issue frequently encountered in Russia, and so a clear explanation of risks and benefits of OCS may be necessary.

When CRSwNP remains uncontrolled despite optimal use of these therapies, patients should then be considered for additional treatment, although it must be emphasized that use of topical irrigation, INS, and intermittent use of OCS [1, 2] remains a critical component of CRSwNP care throughout management [1, 2, 6-8, 26, 27]. In the specialist setting, many patients may have received these therapies before referral, and indeed the need for surgical management may already be evident from CT/endoscopic management on initial assessment. However, it seems prudent to suggest that these medical therapies should be continued under specialist supervision for a short period (eg, up to 8 weeks) before further treatment decisions are made.

For the majority of patients with uncontrolled disease, the preferred option is ESS [4, 5, 40], although for some patients, use of biologics can be considered at this point [26]. The goals of ESS are to remove diseased tissue and improve anatomical function within the paranasal sinus and nasal cavities to optimize access for topical irrigation and steroid delivery [26, 40]. In EPOS 2022, the suggested minimal threshold criteria for surgical intervention are that ESS should be considered in those patients with a LMS of ≥1 on CT, with persistent symptomatic disease, and with a total SNOT-22 score ≥20 after at least 8 weeks of medical therapies, although with a caveat that not all patients meeting these should necessarily have surgery [1, 2]. However, disease recurrence following ESS is high requiring subsequent revision procedures (ranging up to 25%) [41, 42]. Revision rates are even higher in CRSwNP with coexisting asthma [41, 43, 44].

## 9. Use of biologics in CRSwNP

The emergence of effective biologics, chiefly targeting the underlying type 2 inflammatory pathways, provides additional options for patients with refractory or uncontrolled CRSwNP [45, 46]. Globally, a range of agents are approved for use in CRSwNP, although specific approval varies widely across APAC and in Russia. Dupilumab is a human monoclonal IgG4 antibody targeting the IL-4Rα subunit present on the IL-4 and IL-13 receptor complexes to inhibit IL-4 and IL-13 signaling (key and central elements of type 2 inflammatory pathways) leading to reduced IgE production and eosinophil recruitment [14, 45, 47]. Omalizumab (a human monoclonal IgG1 antibody) binds the Fc region of circulating IgE, blocking IgE interaction with mast cells, basophils and B-cells carrying the high-affinity (Fc-ε-RI) IgE receptor, and downregulation of receptor expression by these cells. IgE production is decreased due to the inhibition of mast-cell IL-4 release driving IgE synthesis, and by a direct effect on IgE B-cell production [45]. Mepolizumab is a human monoclonal IgG1 antibody that binds circulating IL-5 to inhibit its interaction with the α-chain on the IL-5 receptor on eosinophils, leading to downstream inhibitory effects on eosinophil maturation, recruitment, and survival [45, 47].

Use of biologics is now established in current guidelines, although differences exist in specific criteria and thresholds [1, 2, 6-8, 27]. The EPOS 2020 guidelines indicate that biologics can be considered if at least 3 of the following 5 features are present; (1) evidence of type 2 inflammation (tissue eosinophilia ≥10 cells/high-power field, or serum eosinophilia ≥250 cells/µL, or total serum IgE ≥100 IU/mL); (2) need for systemic corticosteroids (≥2 courses OCS per year or long-term [>3 months] low-dose steroids) or contraindications for systemic corticosteroids; (3) significantly impaired QoL (eg, with SNOT-22 ≥40); (4) significant loss of smell (anosmia on smell test); (5) with comorbid asthma (requiring regular inhaled corticosteroids) [1, 2]. Looking specifically at the criteria for evidence of type 2 inflammation, in the panel’s view, while evidence of type 2 inflammation is an important criterion, these cutoff thresholds may not be fully appropriate or applicable for Asian populations, and their strict application may obstruct access to patients who may benefit from biologics. For Asian patients, alternative thresholds may apply. Serum eosinophilia ≥250 cells/µL may be the most reliable marker of type 2 inflammation in Asian patients, while a total serum IgE ≥150 IU/mL is more typical of nontype 2 inflammation. Tissue eosinophilia of ≥10 cells/high-power field may be a weak biomarker in Asian patients, where when used to assess status in patients with recurrent disease following surgery, a higher threshold of between 70 and 100 eosinophils per high-power field is used. When considering the need for systemic corticosteroids, patient reluctance or refusal to take systemic steroids should also be taken into account.

The positioning of biologics in the treatment of CRSwNP continues to evolve. At present, EPOS 2020 principally recommends use of biologics for CRSwNP refractory to routine medical therapy, and where disease persists despite previous ESS. However, EPOS 2020 also indicates that biologics can and should be considered in those patients where surgery is contraindicated, and for patients with severe asthma, and/or other comorbidities with surgical risk (eg, significant cardiac disease). In addition, in some cases, patient preference may be not to have surgery [1, 2]. The panel agreed with this approach. At the present time, the panel makes no recommendations for use of specific agents (although most clinical experience in CRSwNP is with dupilumab, in part reflecting its earlier approval for use in CRSwNP patients). Ultimately choice of any specific biologic will be influenced by patient profile, physician experience, and agent availability.

Based upon the earlier criteria developed by EUFOREA [6], specific criteria for assessment of treatment response to biologics are included within EPOS 2020, where 5 objective criteria are considered (reduced nasal polyp size on endoscopy, reduced need for systemic corticosteroids, improved QoL [eg, as measured using SNOT-22], improved sense of smell, and reduced impact of comorbidities) [1, 2]. Response is graded based on the number of these parameters being met; 0 (no response), 1 to 2 (poor), 3 to 4 (moderate), and 5 (excellent). The panel considered these to be useful for the APAC region and Russia but would also wish to emphasize the importance of nasal obstruction as a particular troubling patient symptom (although also recognizing that the impact of nasal obstruction is also captured within endoscopic and SNOT-22 measures).

These criteria are useful to evaluate benefits of a chosen biologic and to assess whether discontinuation and/or switching to an alternative biologic is indicated. While biologics such as dupilumab show evidence of improvement as early as within 4 to 8 weeks [48], in EPOS 2020, assessment after 16 weeks is suggested as an appropriate timepoint for evaluation of initial treatment response [1, 2]. In the panel’s view, a shorter period can be used (eg, at 12 weeks). There are at present relatively limited data for the benefit of switching to an alternative biologic therapy in patients not responding to an initial therapy, and at present the broader panel view is that while alternative biologics could be considered, further ESS should be preferred.

## 10. Educational needs

Changing landscape due to emerging data and reimbursement strategies make standardization of choices difficult at present. This will be an evolving space for some time, and updates will be needed and localized to specific contexts.

## 11. Conclusions

In the present paper, we present key aspects of the current understanding of CRSwNP and provide guidance for physicians within the APAC region and Russia on the clinical management of patients with CRSwNP. Although based upon existing clinical guidelines, a full appraisal of the supportive evidence was beyond the scope of the present paper. The consensus of the panel is that most patients will benefit from a multidisciplinary approach to evaluation and clinical management. Most patients respond to medical treatment (principally focused on topical treatments directed toward inflamed nasal mucosa and adjunctive short-term use of systemic corticosteroids). However, those with more severe/uncontrolled disease require surgery. In this, patient selection is critical (and for some patients, treatment with biologics could be considered at this stage). For those patients with persistent, uncontrolled symptoms despite surgery, biologics should be considered. There remains a need to better characterize CRSwNP patient profiles within the APAC region. Data on the impact of biologics in Asian patients are limited and such data may help in cost-effectiveness evaluations to support use of biologics in the region.

## Acknowledgments

The authors would like to thank Irena Mandic and Stephen McGrath (IntraMed Communications) for their support in facilitating the Expert Panel meetings and subsequent activities. Iain O’Neill (independent medical writer) provided support in manuscript development.

The logistics of the panel selection, meeting facilitation, and subsequent discussions and manuscript development were supported by an unrestricted grant from Sanofi. The sponsor had no influence or involvement in the recommendations developed from the discussions or on the content and viewpoints expressed in this manuscript.

## Conflicts of interest

SK has been a speaker and lecturer for Sanofi, Medtronic, Storz, MSD, Solopharm, Bionorica; has received honoraria for advisory boards from Sanofi, Solopharm, Bionorica, MSD; has participated in clinical trials for Sanofi, Covance, Parexel, and GSK. KS has received grants and/or personal fees from ALK, AstraZeneca, Novartis, Sanofi Genzyme, Seqirus, Stallergenes-Greer; has been on the EAACI board for Allergen Immunotherapy; National Allergy Centre of Excellence (Australia) board for Insect Allergy; has been an advisor to InflaMed Pty Ltd. The remaining authors declare no conflicts of interest.

## Author contributions

Conceptualization: SK, YGJ, D-WK, KS, RK-YT and T-HY; Literature review: SK, YGJ, D-WK, KS, RK-YT and T-HY; Manuscript writing, critical revision, and approval: SK, YGJ, D-WK, KS, RK-YT and T-HY.
